# Vocal repertoire and consistency of call features in the meagre *Argyrosomous regius (Asso*, *1801)*

**DOI:** 10.1371/journal.pone.0241792

**Published:** 2020-11-05

**Authors:** Marta Bolgan, Beatriz P. Pereira, Aurora Crucianelli, Constantinos C. Mylonas, Pedro Pousão-Ferreira, Eric Parmentier, Paulo J. Fonseca, M. Clara P. Amorim

**Affiliations:** 1 MARE - Marine and Environmental Sciences Centre, ISPA - Instituto Universitário, Lisbon, Portugal; 2 Department of Animal Biology, Faculty of Science, University of Lisbon, Lisbon, Portugal; 3 Laboratory of Functional and Evolutionary Morphology (Department of Biology, Ecology & Evolution), FOCUS, AFFISH-RC, Institut de Chimie B6c, University of Liège, Liège, Belgium; 4 Institute of Marine Biology, Biotechnology and Aquaculture, Hellenic Center for Marine Research, Heraklion, Crete, Greece; 5 Instituto Português do Mar e da Atmosfera, Olhão, Portugal; 6 Department of Animal Biology and cE3c- Centre for Ecology, Evolution and Environmental Changes, Faculty of Science, University of Lisbon, Lisbon, Portugal; University of Windsor, CANADA

## Abstract

Passive Acoustic Monitoring (PAM) is a non-intrusive and cost-effective method capable of providing high-resolution, long-term information on the status and health of vocal populations and communities. To successfully monitor the same species over wide geographical and temporal scales, it is necessary to characterise the range of sound variability, as well as the consistency of sound features between populations. The meagre (*Argyrosomus regius*, Asso 1801) is an interesting case study because recent investigations suggest a wider vocal repertoire than previously described. In this study, meagre vocalizations were recorded and analysed from a variety of settings, ranging from rearing facilities to wild populations to provide a comprehensive characterisation of its vocal repertoire, while investigating the consistency of spawning sound features between populations. All sounds presented a similar acoustic structure in their basic unit (*i*.*e*. the pulse), while an important variability was found in the number of pulses; the meagre can emit sounds made of one single pulse or many pulses (up to more than 100). High level of overlap in the Principal Component Analysis made difficult to differentiate sound type clusters. Despite this, two sound types were identifiable: knocks (sounds from 1 to 3 pulses) and long grunts (sounds with more than 29 pulses). Discriminant Analysis carried out on PCA residuals showed that knock had the highest proportion of correct placement (92% of the observations correctly placed) followed by long grunts (80%). All other previously described sound types (intermediate grunt, short grunt and disturbance sounds) could not be separated and presented low levels of correct placement, suggesting that care should be taken when defining these as independent sound types. Finally, acoustic features consistency was found in meagre grunts emitted by different populations during spawning nights; statistical differences could be explained by recording settings and fish conditions. The results of this study provide important information for fostering PAM programs of wild meagre populations, while contributing to the discussion around the definition of fish sound types in vocal fish communities. Studies of this kind, which evaluate both variability and consistency of sound features, are of fundamental importance for maximising PAM efforts in the wild, at both the specific and the community level.

## Introduction

Since the mid-20th century, the rapid increase in human population has resulted in a variety of human-induced changes to the biosphere, the geosphere and the atmosphere [1]. Human activities are destroying biodiversity around 1,000 times faster than natural ‘background’ rates [2] so that the modern loss of species diversity has been labelled as the ‘sixth extinction’ [3]. With ever increasing anthropogenic pressures on ecosystems, the monitoring, management and conservation of wild populations becomes imperative. One of the main challenges is to find non-invasive monitoring methods capable of providing high-resolution, long-term and large-scale information on habitat health and species welfare.

Passive acoustic monitoring (PAM) is a non-intrusive, behaviourally unbiased, cost-effective method with the potential of providing high-resolution, long-term information on ecosystems health [4–7]. In aquatic environments, PAM involves the use of hydrophones to record all components of underwater soundscapes (*i*.*e*. the acoustic scene emanating from a habitat), including abiotic sounds (*e*.*g*. geophysical sources), biological sounds (invertebrates, fish and marine mammals) and human-generated noises (*e*.*g*. shipping, construction, oil and gas exploration) [8–12]. Natural sounds collected using PAM, especially those from vocal animals, can be used as proxies to learn about the diversity of species, habitat quality, the phenology of biological events and the health of fish and shellfish stocks [13].

The use of Passive Acoustics for monitoring fish populations has emerged as a valuable tool in fisheries science [8,11,14,15]. This method has been used to investigate fish presence, distribution, relative abundance, diel, lunar and seasonal cycles of activity, as well as for delimitating spawning areas and for studying wild fish spawning behaviour [8,11,15–22]. Since sound emission is associated with reproduction in many species, this method became especially useful for monitoring spawning sites at both temporal and geographical scales [15–17,19,21,23]. This is particularly true for the family of Sciaenidae, which spawning aggregations have been successfully monitored in the wild thanks to their communicative sounds [15–17,19–23].

On the other hand, studies monitoring patterns of vocal fish communities for inferring long-term information on species richness and ecosystem health are still relatively rare [24,25]. Two main methodological trends are emerging in such studies: the application of acoustic indexes and approaches based on the automatic detection of calls. The use of acoustic indices to unravel complex biophonic patterns is advantageous because it does not require prior knowledge of the targeted signals. However, acoustic indices, such as the Acoustic Complexity Index, cannot discern between fish sound diversity and abundance [26]. In this context, approaches based on manual or automated detection of fish sound types and sequences appear to be the key for describing high resolution fish biodiversity dynamics through passive acoustic monitoring [11,27,28].

Scientists interested in using fish sound types as units for single species monitoring, as well as for monitoring the health of vocal fish communities, face different challenges, such as: *i*) lack of standardized nomenclature for fish sound types, *ii*) reliance on the use of onomatopoeic names, *iii*) limited knowledge about most of the sound types recorded in natural habitats and *iv*) limited knowledge of the range of variability of fish sound types within a single species (*e*.*g*. vocal repertoire) [12,29–31]. Efforts to create a more uniform and objective sound nomenclature and to increase our knowledge on fishes’ acoustic repertoire are therefore warranted. Furthermore, to successfully monitor the same species over wide geographical and temporal scales, it is necessary to account for the range of variability of its acoustic repertoire. Finally, another fundamental requirement for reliable PAM concerns the assessment of call features’ consistency between populations, as demonstrated recently for the large scale and long-term monitoring of the brown meagre (*Sciaena umbra*, Linnaeus 1758) [32].

The common vernacular name of the teleost family of Sciaenidae, *i*.*e*. croakers and drummers, clearly points to their vocal abilities, which have been known and exploited for centuries [33–38]. Several studies have monitored sciaenids and their spawning aggregations in the wild by using PAM [16–17,19–22,32,39–41]. Despite this, sound production in this family has been documented in only about 8% of the extant species; furthermore, lack of consistency can be found in sound types definition and nomenclature, as well as in the type of features used for characterising sounds.

The meagre (*Argyrosomus regius*, Asso 1801) is an interesting case because recent studies [41–43] suggest a wider vocal repertoire than previously described [37]. This species is widely distributed along the Atlantic coast of Europe and of Africa, as well as in the Mediterranean Sea [44] and has a significant commercial value for recreational and small-scale commercial fisheries, as well as aquaculture [45]; this implies that knowledge about meagre sound feature variability and consistency is imperative for populations monitoring over wide geographical and temporal scales. In this study, we characterised the variability of meagre sounds, as well as the consistency of sound features between spawning populations, with the final goal of providing valuable information for maximizing PAM efforts for this species in the wild. Furthermore, our results contribute to the discussion around the definition of “fish sound type”, which is the fundamental unit for monitoring status and health of vocal fish populations by using PAM.

The specific aims of this study were therefore to *i*) characterise the overall acoustic repertoire of the meagre and *ii*) verify if consistency in sound temporal and spectral features can be found in spawning sounds emitted by meagre belonging to two different populations (South Portugal and South France).

## Materials and methods

### Acoustic data collection

In order to provide a comprehensive characterisation of the meagre vocal repertoire (first aim), meagre vocalizations were recorded in a variety of settings, ranging from rearing facilities to wild populations. In particular, advertisement sounds (*i*.*e*. sounds naturally emitted by the fish during social interactions) were recorded in two research aquaculture rearing facilities as well as in the field (Tagus estuary, Portugal). Disturbance sounds (*i*.*e*. sounds emitted by the fish when hand-held) were registered at the rearing facility of the Instituto Português do Mar e da Atmosfera—Estação Piloto de Piscicultura de Olhão (IPMA—EPPO, Portugal). The consistency of calls features (second aim) was assessed for advertisement calls emitted during spawning nights by meagre belonging to two different populations (South Portugal and France), hosted in two aquaculture facilities.

#### Captivity recordings; advertisement sounds

Advertisement sounds were recorded in rearing facilities from two groups of adult meagre breeders belonging to two original populations: at IPMA, fish belonged to the second and the fourth generations of originally wild populations from Southern Portuguese waters. At the Institute of Marine Biology, Biotechnology and Aquaculture—IMBBC, Hellenic Centre for Marine Research (HCMR, Heraklion, Crete, Greece), fish were the third farmed generation of originally wild Mediterranean French populations.

Authorization for scientific experimental work at IPMA was obtained by the Portuguese National Authority for Animal Health, *i*.*e*. DGAV (DGAV reference 0421/000/000/2018), in accordance with European regulations. For the experiments at HCMR-IMBBC, ethical approval for the experimental procedures was obtained from the relevant Greek Authorities (National Veterinary Services, Heraklion, Crete) under the license No 255356 (AΔA:6ΛI17ΛK-ΠΛΩ). The approvals were obtained for the number of fish used and the rearing conditions, handling/sampling (when appropriate). Samplings were always conducted under anaesthesia. Specifically, for the induction of oocyte maturation, ovulation and spawning (see below), fish were initially tranquilized in their tank with the use of clove oil (0.01 ml l^-1^). Then, the fish were transferred to a separate tank for complete sedation with a higher concentration of clove oil (0.03 ml l^-1^) [46]. All procedures conducted in both facilities were in accordance to the “Guidelines for the treatment of animals in behavioural research and teaching” [47], the Ethical justification for the use and treatment of fishes in research [48] and the “Directive 2010/63/EU of the European parliament and the council of 22 September 2010 on the protection of animals used for scientific purposes” [49].

At IPMA, eight sexually mature, 6 and 9-year-old meagre (6 females and 2 males) with a body weight of 4.3 to 9 kg were reared in one 3.6 m^3^ (3 m^2^ area, 1.2 m deep) indoor concrete parallelepipedal tank supplied with filtered seawater which temperature ranged from 14°C to 23°C, and under simulated ambient photothermal conditions (10L/14D hours, with low light intensity at dawn and dusk). The broodstock tank was fitted with passive egg collectors, which were examined frequently during the expected spawning days and, when present, floating eggs were removed, incubated and reared according to protocols developed at IPMA [50]. Sound production was monitored round-the-clock (24 h / day) from February to July 2018. A custom-made hydrophone [51] was positioned vertically in the centre of the tank at approximately 30 cm from the bottom and connected to a stand-alone 16 channel datalogger (LGR– 5325, Measurement Computing Corp, Norton Ma USA; 12 kHz sampling rate 16 bit, ± 1 V range). For the purpose of this manuscript, sound production was inspected during two phases: the first phase represented the pre-spawning period, corresponding to the 5^th^ day prior to the first spawning event (*i*.*e*. 28/04/2018), while the second phase consisted of the spawning period, from the evening that preceded egg deposition to the day after egg deposition (*i*.*e*. 02-03-04/05/2018 mean water T = 19.2 °C; 18-19-20/05/2018 mean water T = 21.8 °C; 04-05-06/06/2018 mean water T = 20.1 °C; 04-05-06-07/07/2018 mean water T = 21.1 °C).

At HCMR, seventeen sexually mature, 5-year-old meagre (11 females and 6 males) with a body weight of 5.0 to 11.3 kg were housed in a 15 m^3^ (6.5 m^2^ area, 2.45 m deep) concrete tank supplied with seawater under simulated ambient photothermal conditions, using heated/cooled water and LED lights of ocean lighting spectrum (Aquaray, TMC, UK). The broodstock tank was fitted with passive egg collectors, which were examined frequently during the expected spawning days/times, when eggs were collected and evaluated under a microscope to estimate fecundity and fertilization success. Sound production was monitored by using an underwater acoustic datalogger (SNAP = hydrophone sensitivity; −170 dB re. 1 V/μPa, Loggerhead Instruments, FL, USA) deployed in the centre of the tank. The SNAP recorded .wav files at 44100 Hz, 16 bits and was set for recording 300 s every 900 s, meaning that the meagre activity was monitored thanks to 3 recordings, each lasting 5 minutes, taken each hour. Sound production was monitored during three phases: reproductive quiescence (5-8/03/2018), pre-spawning period (3-7/05/2018) and spawning period (8-10/05/2018). The reproductive quiescence recordings were carried out under lower water temperature (T = 16 °C) and with less hours of light than during the breeding season. The second phase represented the onset of the breeding season (water T = 20.0 °C), when fish were confirmed to be in reproductive conditions (female contained fully vitellogenic oocytes, > 500 μm in diameter, while males were releasing sperm upon application of gentle abdominal pressure). The last recording session was carried out after the females were induced to undergo oocyte maturation, ovulation and spawning using an injection of gonadotropin releasing hormone agonist (GnRHa) at a dose of 15 μg of GnRHa kg^-1^ body weight. Males were administered an implant containing 350 μg of GnRHa to enhance spermiation [52,53]. Following this protocol, meagre spawned over three consecutive days, beginning 36 h after the hormonal therapy (08/05/2018 to 10/05/2018, water T = 20.0 °C).

#### Captivity recordings; disturbance sounds

Disturbance sounds were recorded at IPMA during July 2018. The procedure started by fasting the same individuals recorded before (N individuals = 8; 4.1 to 10.3 Kg mean body weight) for 24 hours prior to recordings. Water level in the rearing tank was lowered to 1 m to reduce stress and to prevent fish from jumping while being captured. Fish were anaesthetised using 40 ppm of 2-phenoxyethanol (2-PE). After ca. 20 min from the provision of the anaesthetic, anaesthetised individuals were captured with a plastic sleeve bag and transferred in groups of 3 to 200 L plastic circular containers provided with aerated seawater. Fish were here allowed to recover for approximately 15 min. Then, each fish was identified by chip reading and transferred with a sleeve into a second 200 L container (with oxygenated seawater), where individual recordings took place. The fish was kept inside the sleeve and stimulated by pressing the caudal peduncle; disturbance sounds were recorded for 3 min using a High Tech 94 SSQ hydrophone (High Tech Inc., Gulfport, MS, USA; sensitivity of –165 dB re 1 V/μPa, frequency response up to 6 kHz within ± 1 dB), placed at approximately 10 cm from the fish’s abdomen, and connected to a Tascam DR-40 Portable Digital Recorder (44.1 kHz sampling rate, 16 bit; TEAC, Europe Gmbh).

#### Field data collections

Meagre vocal activity was monitored during summer 2018 in the Tagus estuary (Air Force base 6, Montijo, Portugal; 38°42'N, 8°58'W). Sound recordings were obtained from wild adult fish of unknow size, sex ratio and group size. A High Tech 94 SSQ hydrophone (High Tech Inc., Gulfport, MS, USA; sensitivity of –165 dB re 1 V/μPa, frequency response up to 6 kHz within ± 1 dB) was anchored at about 20 cm from the bottom to a stainless-steel holder projecting from a concrete base where the cable was attached to minimise current-induced hydrodynamic noise. The signal from the hydrophone was recorded (4 kHz, 16 bit resolution) by a 16 channel stand-alone data logger (Measurement Computing Corporation LGR-5325, Norton, Virginia, USA). Water depth varied approximately between 3 to 6 m, depending on tide. For the purpose of this study, and because meagre sounds in the field recordings were produced in dense choruses in which individual calls could not be distinguished, sounds with a good Signal-to-Noise Ratio (SNR) emitted before or after the chorus were randomly extracted from one day in March (17/03/2018), one day in April (16/04/2018, mean water T = 15.6 °C), one day in May (18/05/2018, mean water T = 19.3 °C), three days in June (15/06/2018, mean water T = 20.0 °C; 16/06/2018, mean water T = 20.0 °C; 17/06/2018, mean water T = 20.4 °C) and one day in August (15/08/2018, mean water T = 22.0 °C).

### Acoustic data analysis

The audio files were analysed by visual and aural inspection by using Raven 1.5 for Windows (Bioacoustic Research Program, Cornell Laboratory of Ornithology, Ithaca, NY, USA). All audio files were downsampled at 4 kHz and spectrograms were visualized using a 64 points FFT, Hanning window. For each situation (*i*.*e*. *advertisement sounds IPMA*, *advertisement sounds HCMR*, *disturbance sounds*, *and field recordings*), a sub-sample of sounds with good SNR was analysed (N = 741 sounds). Spectral features were measured from power spectra (4 kHz, FFT size 512 points, Hanning window, 50% time overlap) while temporal parameters were measured from oscillograms. The following sound features were measured for all sounds: *i)* peak frequency (Peak Freq., Hz; the frequency with the highest energy), *ii)* Q3 frequency (Q3 Freq., Hz; the frequency that divides the spectral content into two intervals containing 75% and 25% of the energy), *iii)* Q1 frequency (Q1 Freq., Hz; the frequency that divides the spectral content into two intervals containing 25% and 75% of the energy), *iv)* sound duration (ms; the time from the onset of the first pulse to the offset of the last pulse), *v*) number of pulses and *vi)* pulse period (ms; the time interval between the peaks of two consecutive pulses in a sound). All pulse periods within each sound were manually measured (N = 20,736 measured pulses).

To evaluate the variability in number of pulses in all analysed sounds, a histogram depicting the proportion of sounds (%, N = 741) having a specific number of pulses was plotted. To assess pulse period variability within each sound, pulse period was plotted against pulse succession. Mean and standard deviation were calculated for all acoustic features by categorising sounds on the basis of their number of pulses, following the classification proposed by [41]. In particular, knocks were sounds made of 1 to 3 pulses, short grunts were sounds ranging from 4 to 6 pulses, intermediate grunts were sounds made of 7 to 29 pulses and long grunts were sounds with more than 30 pulses.

### Statistical analysis

Statistical analysis was performed by using STATISTICA (version 10, Statsoft, Tulsa, OK, USA) and Minitab (version 18, Minitab LLC, State College, Pennsylvania, USA). To examine if independent and mutually exclusive sound classes could be identified, a Principal Component Analysis (PCA) was performed in Minitab 18 on all sounds analysed as part of this study (N = 741). All acoustic features were firstly inspected for correlation; many acoustic features were found to be highly correlated and PCA was performed by using only Q1 Frequency, Peak frequency, Q3 frequency, number of pulses and pulse period as variables. The correlation matrix was used to calculate the principal components, as variables had different scales and they needed to be standardised. A scree plot for the first five components and a score plot for the first two components (which cumulatively explained 66.5% of the variance) were generated. A linear discriminant analysis was run on the PCA residuals to validate the adequacy of sound type classification.

To examine the probability of transition between the sound categories proposed for meagre by [41], a total of 40 sequences made of 20 sequential sounds (total of 720 transitions) were randomly selected between all datasets except for disturbance sounds. As an example, the scoring of a sequence produced this kind of string: long grunt→intermediate grunt→long grunt→long grunt and so on. Data were entered in contingency tables, with preceding sound categories in rows and immediately following sound categories in columns. Chi-square analysis at 0.001 level of significance was run within these contingency tables. Cells with residuals of chi-square exceeding the chosen level of significance were identified to detect the sequences that occurred more (or less, if the residuals were negative) frequently than predicted by chance [54]. Sequence analysis of sound types was represented graphically in flow diagrams. Each sound type was outlined as a circle, and sequences between preceding and immediately following sound types were shown by connecting these circles with arrows proportional in width to magnitude of the chi-square residuals.

The consistency of acoustic features of sounds emitted by the two genetically distant meagre populations (South Portugal and Mediterranean Sea, France [55]) was examined by comparing only sounds emitted during spawning nights to minimize spurious variability (N = 244 sounds). Analysis of Covariance (ANCOVA) was performed on all measured acoustic features, using the aquaculture facility as factor (IPMA or HCMR) and water temperature as a covariate, as temperature has been proven to influence sound features in animals such as fish, which are poikilotherms [41,56,57]. ANCOVA was run on all sounds considered together, as well as on sounds assigned to different categories on the basis of their number of pulses (short grunts, intermediate grunts and long grunts). To meet ANOVA assumptions, data were log_10_-transformed prior to the ANCOVA analysis.

## Results

**Fig 1:**
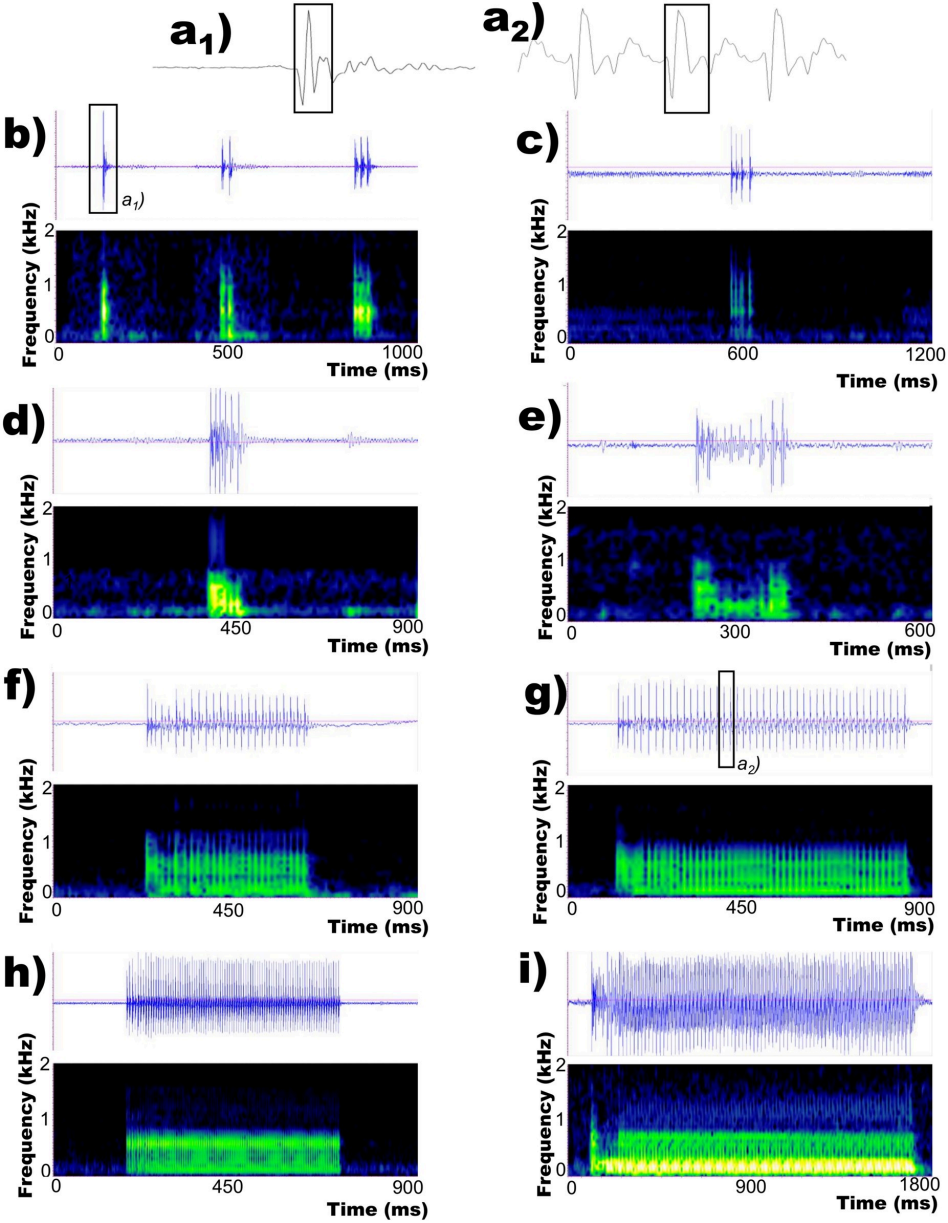
Oscillograms and spectrograms representing the variability of *Argyrosomus regius* vocal repertoire: a) pulse waveform (a1 = pulse waveform of 1 pulse knock, see rectangle in b; a2 = pulse waveform within a long grunt, see rectangle in g); b) knocks (1, 2 and 3 pulses); c) short grunt (4 pulses); d) short grunt (6 pulses); e) intermediate grunt (11 pulses) with a typical pattern in amplitude modulation; f) intermediate grunt (24 pulses); g) long grunt (44 pulses) with limited amplitude modulation; h) long grunt (87 pulses) and i) long grunt (100 pulses) with a typical pattern in amplitude modulation. Sampling frequency 4 kHz, 64 point FFT (frequency resolution: 63 Hz), Hamming window, 50% overlap.

*Argyrosomous regius* emitted pulsed sounds with most energy below 1 kHz (Fig 1), where for pulse we mean a sharp increase in acoustic energy which amplitude decays rapidly [31].

**Fig 2:**
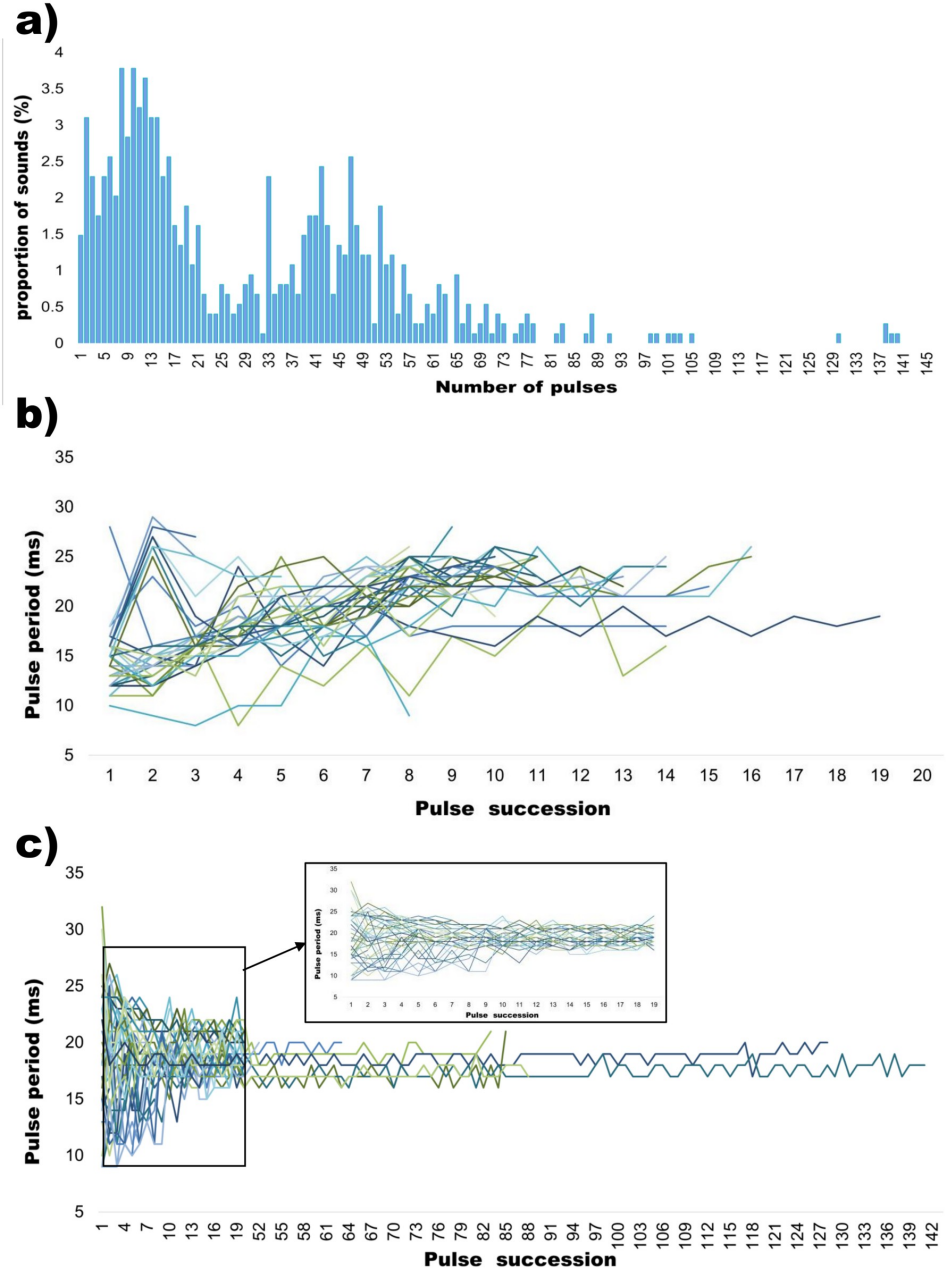
a) Number of pulses (proportion of sounds, %) in *Argyrosomus regius* sounds (all sounds analysed as part of this study, i.e. N = 741); b) pulse period succession in grunts having less than 20 pulses and c) pulse period succession in grunts having more than 20 pulses (the box on the upper right of panel c represents a zoom on the first 20 pulses).

Although all sounds presented a similar acoustic structure in their basic unit, *i*.*e*. the pulse (Fig 1a, S1 and S2 Figs), the number of pulses within a single sound presented an important variability (Figs 1 and 2a).

The meagre can indeed emit sounds made of one single isolated pulse, few pulses, or many pulses up to more than 100 pulses (Figs 1 and 2a, Table 1). In sounds consisting of more than a few pulses, amplitude modulation (*i*.*e*. change in the amplitude level of the soundwave over time) can be present (Fig 1e), but it is generally restricted to the first pulses within the call (*e*.*g*. Fig 1f and 1i). Pulse period succession was rather regular within the same sound, where most of the pulse period variability occurred within the first pulses (Fig 2b and 2c). After this first initial variability, pulse period showed an extremely reduced range of variation even in very long sounds (more than 100 pulses) (Fig 2c).

**Table 1:** Descriptive statistics (mean, standard deviation SD and coefficient of variation CV) of sound features characterising the calls emitted by the meagre *Argyrosomus regius*. All sounds analysed are pooled together. Q3 Freq = Q3 frequency; Peak Freq = peak frequency; Q1 Freq = Q1 frequency.

Context		Disturbance	Advertisement			
Sound category		Disturbance	Knock	Short grunt	Intermediate grunt	Long grunt
Sample size		46	50	50	270	325
**Q3 Freq (Hz)**	Mean	373.1	566.8	478.6	416.4	413.7
	S.D.	88.7	45.0	127.9	106.9	98.7
	C.V.	23.8	7.9	26.7	25.7	23.8
**Peak Freq (Hz)**	Mean	232.2	437.7	331.0	262.8	231.6
	S.D.	73.3	112.6	158.0	125.3	125.4
	C.V.	31.6	25.7	47.7	47.7	54.1
**Q1 Freq (Hz)**	Mean	220.3	375.6	254.5	213.6	197.8
	S.D.	60.7	58.7	86.2	66.1	87.5
	C.V.	27.6	15.6	33.8	30.9	44.2
**Duration (ms)**	Mean	169.6	79.3	126.8	271.6	957.3
	S.D.	72.8	161.6	134.6	113.7	323.1
	C.V.	42.9	203.7	106.2	41.8	33.8
**Number of pulses**	Mean	11.6	2.1	5.1	14.8	51.2
	S.D.	3.1	0.7	0.8	5.7	17.9
	C.V.	27.1	34.9	15.8	38.5	35.0
**Pulse period (ms)**	Mean	14.5	29.4	18.3	17.8	18.9
	S.D.	2.9	12.6	4.0	3.2	2.2
	C.V.	19.7	43.0	21.7	17.9	11.9

In knocks made of more than 1 pulse, pulse period was on average longer than in grunts (Table 1). This explains why knocks and grunts sound different when auditioned, despite of their similarity in pulse envelope (Fig 1a, S2 Fig). Grunts’ pulse period is rather quick in both disturbance and advertisement sounds (Table 1), so that these sounds are heard as a unit (onomatopoeically “grunt”) rather than a train of individual pulses.

Principal Component Analysis carried out on all analysed sounds (N = 741) showed that 66.5% of the variance was explained by spectral features such as peak and Q1 frequency, and temporal features such as number of pulses and pulse period (Table 2).

**Table 2:** Principal Component Analysis (PCA) carried out on all *Argyrosomus regius* sounds; relevant coefficients, eigenvalues, percentage of the variance and cumulative percentage of the variance explained by the first five PCA. Q3 Freq = Q3 frequency; Peak Freq = peak frequency; Q1 Freq = Q1 frequency. The main contributors to the first two components are highlighted.

Variable	PC1	PC2	PC3	PC4	PC5
**Q3 Freq (Hz)**	0.443	0.075	-0.37	-0.813	-0.02
**Peak Freq (Hz)**	**0.557**	0.015	-0.228	0.425	-0.676
**Q1 Freq (Hz)**	**0.577**	0.015	-0.109	0.347	0.731
**Number of pulses**	-0.269	**0.817**	-0.485	0.148	0.053
**Pulse period (ms)**	0.298	**0.571**	0.751	-0.126	-0.075
**Eigenvalue**	2.4	0.9177	0.845	0.6155	0.214
**Percentage**	48.2	18.4	16.9	12.3	4.3
**Cumulative %**	48.2	66.5	83.4	95.7	100

**Fig 3:**
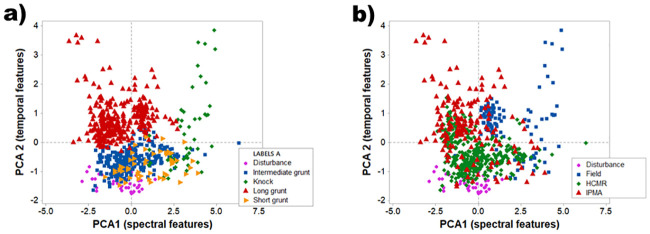
Score plot of the Principal Component Analysis (PC1 and PC2) carried out on all *Argyrosomus regius* sounds analysed as part of this study (N = 741). Variables: Q3 Frequency, peak frequency, Q1 frequency, number of pulses and pulse period (correlation matrix). a) Colour coding indicates sound types categories; b) colour coding indicates recording settings and conditions.

When considering sound type categories (Fig 3a), the score plot of the first two principal components showed very high level of overlap, especially between short, intermediate grunts and disturbance sounds (Fig 3a). Overall, only 62% of sounds were placed into the correct sound type category by the discriminant function analysis; knock had the highest proportion of correct placement (92% of the observations correctly placed) followed by long grunts (80%) (DFA, d.f. = 8, P <0.001, Table 3).

**Table 3:** Classification summary of the discriminant function analysis (linear) carried out on the first five PCA (N sounds = 741).

	True groups				
Put into groups	Disturbance sounds	Intermediate grunts	Knocks	Long grunts	Short grunts
Disturbance sounds	18	75	0	45	4
Intermediate grunts	18	108	0	16	12
Knocks	0	2	45	0	4
Long grunts	3	11	0	259	1
Short grunts	7	74	4	2	30
**Total N**	46	270	49	325	51
**N correct**	18	108	45	259	30
**Proportion**	0.391	0.400	0.918	0.802	0.588

Very high overlap could also be observed in the score plot of the first two principal components when considering recording settings and locations (Fig 3b); this, in its turn, suggests consistency of call features between settings and populations.

**Fig 4:**
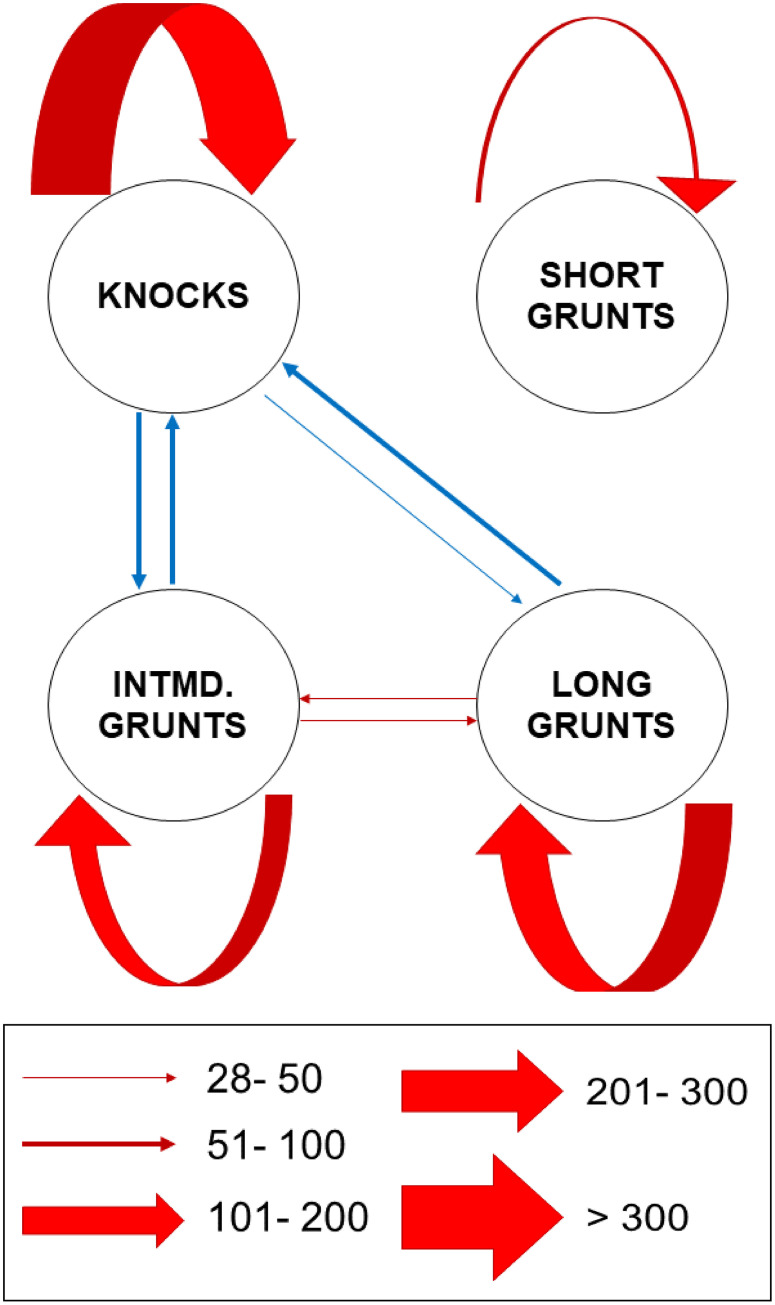
Flow diagram of sound sequences exhibited by *Argyrosomus regius* (X^2^ = 1198.7, df = 9, p.value< 0.001). Red arrows indicate positive transitions that were highly significant (p.value< 0.001). Blue arrows indicate significant negative transitions (p.value< 0.001). Arrows are sized according to the chi-square residual values.

Sequence analysis showed that knocks, long grunts and intermediate grunts tend to be produced in repetitive sequences more often than predicted by chance in the examined datasets (*i*.*e*. advertisement sounds recorded at IPMA and HCMR and field recordings, X^2^ = 1198.7, df = 9, p.value < 0.001; Table 4; Fig 4).

**Table 4:** Results of the sequence analysis carried out on *Argyrosomus regius* sound categories. Significant transitions are highlighted (level of significance = 0.001).

	**Antecedent**				
**Subsequent**		**knock**	**short grunt**	**intermediate grunt**	**long grunt**
***Observed- Expected***	**Knock**	151.3379	-16.0627	-80.0244	-55.2508
	**short grunt**	-16.5335	23.87885	7.139214	-14.4846
	**intermediate grunt**	-77.0956	4.225292	133.9692	-61.0988
	**long grunt**	-57.7088	-12.0414	-61.084	130.8342
**Chi-Square residuals**	**Knock**	**443.3267**	15.12131	**74.44293**	**44.72696**
	**short grunt**	14.7493	**93.1524**	1.651558	8.568792
	**intermediate grunt**	**70.67831**	0.642782	**128.1699**	**33.60132**
	**long grunt**	**52.27389**	6.890991	35.17261	**203.3795**

Interestingly, long sequences of knocks (which could last hours) were recorded in the field and during spawning nights at IPMA, but they were never recorded during spawning nights at HCMR. At both aquaculture facilities, spawning nights were characterised by long sequences of numerous and loud long grunts. In the field, dense choruses of grunts, lasting for hours, have been recorded. In more rare cases, sequences of long and intermediate grunts were interspaced with knocks; this explains the negative transitions between knocks, intermediate and long grunts which can be observed in the flow diagram (Fig 4).

Finally, consistency of call features was found in grunts emitted during spawning nights in both aquaculture facilities. No statistical difference was found in any of the measured acoustic features when considering all grunts together and when controlling for the effect of water temperature on sonic muscles and central pattern generator (Table 5). When considering short, intermediate and long grunts separately, statistical differences between aquaculture facilities were found only in Q3 frequency of intermediate and long grunts, and in long grunts temporal features (Table 5).

**Table 5:** Results of the one-way analysis of covariance (ANCOVA) carried out for comparing grunts emitted by *Argyrosomus regius* during spawning nights in the two aquaculture facilities (IPMA and HCMR) while controlling for the effect of water temperature (covariate). SS = Sum of Squares; MS = Mean Square; Df = degree of freedom. Q3 Freq = Q3 frequency; Peak Freq = peak frequency; Q1 Freq = Q1 frequency.

	Q3 Freq (Hz)	Peak Freq (Hz)	Q1 Freq (Hz)	Duration (ms)	Pulse period (ms)	Number of pulses
**All grunts considered together**						
**Df Model**	1	1	1	1	1	1
**Df Residual**	236	236	236	236	236	236
**F**	0.148	2.499	1.781	1.015	0.320	1.754
**p-value**	0.700	0.115	0.183	0.315	0.572	0.187
**Short grunts**						
**Df Model**	1	1	1	1	1	1
**Df Residual**	8	8	8	8	8	8
**F**	0.007	1.564	0.014	0.011	2.208	0.692
**p-value**	0.934	0.246	0.908	0.918	0.176	0.430
**Intermediate grunts**						
**Df Model**	1	1	1	1	1	1
**Df Residual**	81	81	81	81	81	81
**F**	7.40	0.31	0.60	0.40	0.21	3.05
**p-value**	0.01*	0.58	0.44	0.53	0.65	0.08
**Long grunts**						
**Df Model**	1	1	1	1	1	1
**Df Residual**	148	148	148	148	148	148
**F**	4.098	0.384	1.016	16.378	32.503	56.070
**p-value**	0.045*	0.536	0.315	0.000*	0.000*	0.000*

## Discussion

In accordance with recent studies [41–43], it was here demonstrated that the meagre vocal repertoire is wider than previously thought [37]. Furthermore, fine acoustic features of grunts emitted during spawning nights are overall consistent between Portuguese and Mediterranean French populations [55].

### Acoustic repertoire

The meagre emitted pulsed sounds with most energy below 1 kHz ranging from one to more than 100 pulses. These sounds can present modulation of the peak to peak amplitude of the pulse, as well as pulse period variability, typically in the first pulses after which both features become stable. The meagre shares the same sound-producing mechanisms of other sciaenids [37]. Fishes from this family produce sounds thanks to high-speed sonic muscles originating from the hypaxial musculature, surrounding bilaterally the swimbladder and inserting on a central tendon dorsal to the swimbladder [58–60]. Fast contractions of these sonic muscles drive the swimbladder in a transient response, where each muscle-twitch corresponds to one pulse within the call [61]. The similarities in pulse structure between knocks and grunts (Fig 1) suggest that both sounds are produced by the same mechanism. The amplitude variability frequently exhibited in the initial pulses of the meagre grunts might be related to the time required by the sonic muscles to attain the necessary tension [62]. The production of acoustic signals involves the sound producing apparatus itself, as well as the associated nervous system that controls this apparatus [63,64]. As such, the pulse period variability observed at the beginning of grunts may be related to the time that sonic muscles require to “tune” on a specific contraction rate or may be dependent on the central pattern generator. Another possible explanation, as suggested by [41], is that the bilateral coordination of the sonic muscles might alternate in the beginning of the sound to become later synchronized. Furthermore, differences in neuronal firing could also explain the difference in number of pulses between knocks and grunts. Further studies are required for shading light on how the neuronal circuitry underlying sound production influences amplitude modulation, pulse rate and pulse number of meagre sounds.

Does the meagre produce different sound types? And if so, how many? In bioacoustic studies, sound types (or call types) are defined as a category of vocalizations emitted in a particular social context and having a particular function (reproductive, aggressive, defensive) [31]. While terminology is well established and relatively standardised when referring to anuran vocalizations, different terms and different definitions of the same term have been used for other animals, such as birds, insects and mammals [31]. In fishes, this problem is further exacerbated by the tendency of describing sound types with onomatopoeic names such as ‘hum’, ‘grunt’ and ‘growl’, which are extremely subjective [30,31]. In Sciaenidae, the variability of sound type nomenclature and definition is quite extensive, but two main trends can be identified; that following onomatopoeic definition and that discriminating sounds on the basis of the broad context of emission, *i*.*e*. disturbance calls (induced by hand-handling the fish) and advertisement calls emitted during social interactions. Here we tried to evaluate the validity of sound categories using both classifications (onomatopoeic and context of emission). Our study supports that the vocal repertoire of the meagre is wider than previously thought [37], in accordance with more recent studies [41–43]. Lagardère and Mariani [37] reported the presence of short grunts (pulsed sounds ranging from 4 to 6 pulses) and of long grunts (pulsed sounds of more than 30 pulses). However, sounds ranging from 7 to 29 pulses (labelled intermediate grunts by [41]) as well as isolated pulses (knocks) were very abundant in our study. Unfortunately, a precise behavioral association with sound emission is not yet possible for the meagre. The high overlap observed for distress call, short grunts and intermediate grunts in the Principal Component Analysis implies that a cautious approach should be taken when defining meagre sound types on the basis of number of pulses only. The risk is that of classifying a continuum into an artificial discrete set of categories that may not be related with discrete behavioural information and significance. On the other hand, the Principal Component Analysis showed some degree of differentiation in the cases of knocks and long grunts; this was confirmed by the Discriminant function Analysis, as both knocks and long grunts had very high proportion of correct placement. Furthermore, sequence analysis showed that knocks and long grunts occur in self-isolated bouts with significantly higher probabilities than those predicted by chance. This suggests that knocks and long grunts may be associated with discrete behavioural information and significance.

Although our study cannot conclusively claim a specific number of sound types for this species (as behavioral association was not tested), it has nevertheless proved that the variability of pulsed sounds is extremely wide. Pereira et al. [42] have shown that sound features of meagre advertisement and disturbance calls vary with size, sex and age while Bolgan et al. [43] showed that longer grunts, made of a higher number of faster repeated pulses are emitted during spawning. This implies that important biological information (such as age, sex and behavioral state) might be encoded in sound temporal features, such as number of pulses, pulse repetition rate and sound repetition rates.

### Consistency of sound features between populations—Implications for single species PAM

The present study showed an important overall geographical consistency of spawning grunts emitted by captive meagre originating from different populations [55]. When considering the Principal Component Analysis results, it appears evident that sounds recorded in different settings and emitted by different populations present a very high level of overlap, strongly suggesting that meagre sounds provide a reliable tool for monitoring different meagre populations in the wild (Fig 3b). A similar conclusion can be drawn from the ANCOVA results on sounds emitted during spawning nights in the two aquaculture facilities. No statistical difference was found in any of the measured acoustic features when considering all grunts together. When considering short, intermediate and long grunts separately, statistical differences between aquaculture facilities were found only in Q3 frequency of intermediate and long grunts, and in long grunts temporal features. As already shown in previous studies on sciaenid sound features’ consistency [32], these statistical differences can be explained when considering recording conditions. In particular, the high variability of conditions between our datasets, which includes differences in size, number of individuals, sex ratio and hormonal treatments can explain this partial variability. Pereira et al. (2020) found that Q3 frequency is dependent on individual size [42]; fish at HCMR were bigger than fish at IPMA, which may explain this statistical significance. When it comes to temporal features, Bolgan et al. [43] showed that meagre sound duration, number of pulses and pulse period are influenced by spawning motivation; this is in accordance with what was found in other Sciaenidae species, in aquaculture as well as at sea [43,65–67]. At HCMR, spawning was artificially induced by injecting gonadotropin releasing hormone agonist (GnRHa), while fish at IPMA spawned naturally. It is possible that the long grunts temporal features were influenced by hormonal treatments and spawning motivation. It has to be considered that, while grunts were recorded across all datasets (IPMA, HCMR and field recordings), knocks were never recorded at HCMR. The specific influence of different hormonal conditions and of hormonal concentration on meagre sounds deserves, therefore, to be further investigated. On the other hand, fish at HCMR belonged to an original French, Mediterranean population; Lagardère and Mariani did not record knocks in the Gironde estuary either (French, Atlantic population) [37]. The possibility that the presence of knocks is expression of some degree of geographical variability between the repertoire of different meagre populations warrants future investigations.

Although the specific contribution of geographical, hormonal, motivational and individual differences on meagre repertoire and acoustic features cannot be fully detangled, this study showed an important overall geographical consistency of spawning grunts. It has been already highlighted that, when it comes to fish vocalizations, the rigor of statistical purity, while ideal, should be relaxed; consistency can indeed be found despites of minor statistical differences [32,68]. Our findings are in accordance with what was found for the brown meagre, another vocal sciaenid [32]. PAM was proved to be a reliable tool for monitoring brown meagre populations over very long periods of time (*i*.*e*. 17 years) and over wide geographical scales due to the consistency of this species call features (despite of minor statistical differences) [32]. The authors argued that the geographical and temporal consistency in sound features results from constraints associated with the sound-producing mechanism [32]. Recently, Vieira et al. [41] have shown that meagre’s acoustic activity can be successfully monitored over long periods of time by using an automatic pattern recognition technology based on the hidden Markov Model (with accuracy of almost 80%). Also, Monczak et al. [21] have revealed that the long-term PAM of Sciaenidae populations can be used in the wild to eavesdrop on species-specific spawning seasons. Taken together, the consistency of meagre sounds and the advances made in automatic detection support the use of long-term PAM for monitoring this species in the wild over wide temporal and geographical scales. Also, the monitoring of fine acoustic features (such as number of pulses), as well as of sound sequences, could provide valuable, high-resolution information on the behaviour and reproductive state of this species [43].

### Conclusive remarks—Implications for vocal fish communities monitoring

The results of this study partially contribute to the discussion around the definition of “fish sound type” in vocal fish communities. In studies which use the “sound types” as a unit, dichotomous branching of sound types based on fine acoustic features has been successfully applied in both marine and freshwater environments [27,69]. In particular, richness, diversity and abundance of fish sound types revealed a strong relationship with taxonomic diversity in the Mediterranean Sea [27], thus potentially informing on habitat health. One of the widest unidentified sound type categories is that labelled as “pulse series”, sound category within which meagre sounds would fall in. A major problem faced by scientists when analysing the “pulse series category” in communities in which the emitter (and therefore its repertoire) is unknown, is to decide at which level the subdivision of this category should stop. This decision is particularly important if the number of subcategories (*i*.*e*. sound types) is then used for characterising taxonomic diversity. Our study shows that the intra-variability of this fish sound type category can be extremely wide even if only one vocal species is present, and proves the importance of this kind of studies not only in the frame of single species monitoring, but also in the frame of the relatively new, continuously developing field of vocal fish community ecology. Finally, it is possible that high variability within the “pulse series” sound category informs on species welfare; if specific behavioural significances are linked with number of pulses, a high variability might highlight an extensive diversity of behaviours, contexts, age classes and sizes [42,43], which are all crucial for the stability of species and of populations. Together with inter-specific comparisons of sympatric species’ vocal repertoire, the evaluation of both variability (*e*.*g*. vocal repertoire) and of consistency of sound features within the same species is of fundamental importance for maximizing PAM efforts in the wild, both at the specific and at community level. Further studies addressing the intra-specific variability range of fish pulsed sounds and linking these metrics with population health are strongly encouraged.

## Supporting information

S1 Fig Oscillogram of meagre pulses (within grunts) recorded in different conditions. Oscillograms of seven consecutive pulses within grunts recorded in the field and in captivity (HCMR and IPMA).
(TIF)

S2 Fig Oscillogram of meagre pulses (knocks and short grunts) recorded in different conditions. Oscillograms of knocks recorded in the field and of a short grunt recorded in captivity (HCMR).
(TIF)
