# Correction: Vocal repertoire and consistency of call features in the meagre *Argyrosomous regius (Asso*, *1801)*

**DOI:** 10.1371/journal.pone.0294105

**Published:** 2023-11-02

**Authors:** Marta Bolgan, Beatriz P. Pereira, Aurora Crucianelli, Constantinos C. Mylonas, Pedro Pousão-Ferreira, Eric Parmentier, Paulo J. Fonseca, M. Clara P. Amorim

There are errors in the Funding section. The correct Funding statement is: This study was funded by the European Regional Development Fund (FEDER) through the Lisbon’s Regional Operational Programme (LISBOA-01-0145-FEDER-029662) and the Portuguese Foundation for Science & Technology (FCT) through the project FishNoise (PTDC/BIA-BMA/29662/2017). FCT also supported the project PTDC/BIA-BMA/30517/2017, and the strategic projects UID/MAR/04292/2019 granted to MARE and UID/BIA/00329/2019 granted to Centre for Ecology, Evolution and Environmental Changes. The funders had no role in study design, data collection and analysis, decision to publish, or preparation of the manuscript.
