# Supplementary figures and images for: Vocal repertoire and consistency of call features in the meagre Argyrosomous regius (Asso, 1801)
Source: PLoS One. 2020 Nov 5;15(11):e0241792. doi: 10.1371/journal.pone.0241792 (PMC7643951; doi:10.1371/journal.pone.0241792)

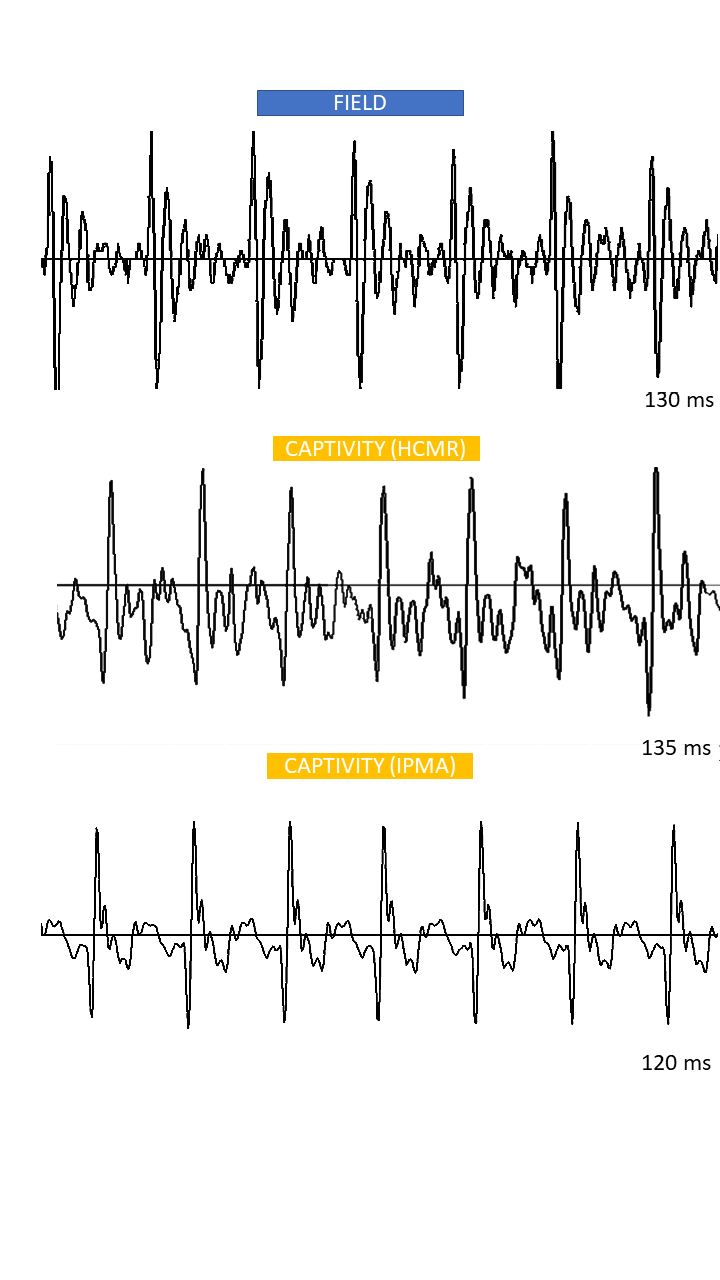

Supplement: S1 Fig — Oscillograms of seven consecutive pulses within grunts recorded in the field and in captivity (HCMR and IPMA). (TIF) [file pone.0241792.s001.tif]

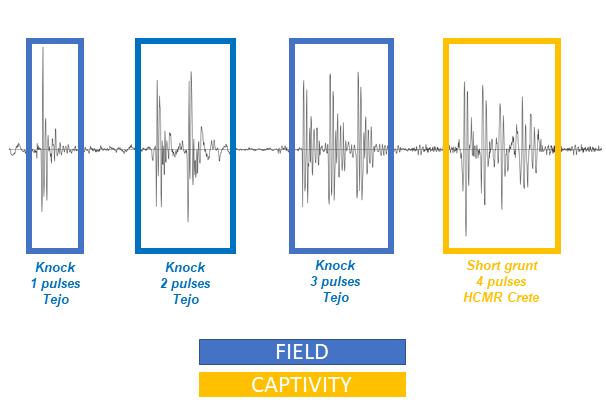

Supplement: S2 Fig — Oscillograms of knocks recorded in the field and of a short grunt recorded in captivity (HCMR). (TIF) [file pone.0241792.s002.tif]
